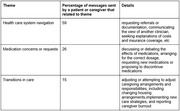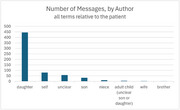# Using qualitative analysis of secure messages to explore the experience of patients with dementia and their caregivers

**DOI:** 10.1002/alz70858_107755

**Published:** 2025-12-26

**Authors:** Lily N Shapiro, Marlaine Figueroa Gray, William I Bowers, Tiina Maripuu, Jenna Mae Peterson, Kelly Ehrlich, Sundary Sankaran, Robert B Penfold, Paul K Crane, Janelle S Taylor

**Affiliations:** ^1^ Kaiser Permanente Washington Health Research Institute, Seattle, WA, USA; ^2^ University of Toronto, Toronto, ON, Canada; ^3^ Department of General Internal Medicine, University of Washington School of Medicine, Seattle, WA, USA

## Abstract

**Background:**

Dementia caregiving presents unique challenges, particularly as family members, who provide the majority of unpaid care and support, navigate complex and unwieldy healthcare systems to secure care. Secure messaging portals are now the primary way patients and their caregivers communicate with healthcare providers outside of structured appointments.^1^ Messages sent through these portals can offer insight into relational and logistical realities of caregiving.

**Method:**

We identified a cohort of adults aged ≥ 65 years diagnosed with dementia from the Adult Changes in Thought (ACT) Study, a prospective cohort study of older adults in Seattle, WA. We compiled a corpus of secure messages sent from participants’ patient portals in the period spanning 5 years before until 5 years after dementia onset. We used Natural Language Processing (NLP) to select messages focused on patients’ lived experiences. We qualitatively analyzed 1023 secure messages between patients or caregivers and healthcare providers to identify themes.

**Results:**

We found several key themes (Table) in the secure messages that relate to caregiving and lived experience. These themes included: 1) healthcare system navigation, 2) medication concerns or requests, and 3) transitions in care.

Only about 15% of messages sent from the patient portal were authored by patients themselves; the rest were authored by individuals in a variety of relationships to the patient; most were family caregivers, primarily daughters (Figure). Traces of the longitudinal, lived experience of patients with dementia and their caregivers come across quite clearly as messages included detailed descriptions of daily life and expressions of emotions such as gratitude, grief, and frustration.

**Conclusion:**

Secure messages initiated through the patient portal provide a valuable window onto the complex social and institutional dynamics of dementia care. These messages, authored by caregivers and/or patients in real‐time and in their own words, reveal difficulties experienced and strategies used to manage both the challenges of both progressive dementia and of dementia care within a complex and fragmented healthcare system.

**Reference**

1. Han, H. R., Gleason, K. T., Sun, C. A., Miller, H. N., Kang, S. J., Chow, S., … Bauer, T. (2019). Using patient portals to improve patient outcomes: systematic review. JMIR human factors, 6(4), e15038.